# Hematologic Manifestations in Celiac Disease—A Practical Review

**DOI:** 10.3390/medicina55070373

**Published:** 2019-07-15

**Authors:** Daniel Vasile Balaban, Alina Popp, Florentina Ionita Radu, Mariana Jinga

**Affiliations:** 1 “Carol Davila” University of Medicine and Pharmacy, 020021 Bucharest, Romania; 2 Gastroenterology Department, “Dr. Carol Davila” Central Military Emergency University Hospital, 010825 Bucharest, Romania; 3 Pediatrics Department, “Alessandrescu-Rusescu” National Institute for Mother and Child Health, 020395 Bucharest, Romania; 4 Faculty of Medicine and Health Technology, Tampere University and Tampere University Hospital, 33100 Tampere, Finland; 5 Faculty of Medicine, Titu Maiorescu University, 004051 Bucharest, Romania

**Keywords:** celiac disease, anemia, lymphoma, IgA deficiency

## Abstract

Celiac disease (CD) is a systemic autoimmune disease driven by gluten-ingestion in genetically predisposed individuals. Although it primarily affects the small bowel, CD can also involve other organs and manifest as an extraintestinal disease. Among the extraintestinal features of CD, hematologic ones are rather frequent and consist of anemia, thrombocytosis (thrombocytopenia also, but rare), thrombotic or hemorrhagic events, IgA deficiency, hyposplenism, and lymphoma. These hematologic alterations can be the sole manifestation of the disease and should prompt for CD testing in a suggestive clinical scenario. Recognition of these atypical, extraintestinal presentations, including hematologic ones, could represent a great opportunity to increase the diagnostic rate of CD, which is currently one of the most underdiagnosed chronic digestive disorders worldwide. In this review, we summarize recent evidence regarding the hematological manifestations of CD, with focus on practical recommendations for clinicians.

## 1. Introduction

Celiac disease (CD) is a chronic, autoimmune condition triggered by gluten ingestion in genetically susceptible individuals. It can develop at any time throughout the life of individuals carrying the predisposing DQ2/DQ8 haplotype, leading to a gluten-dependent small-bowel inflammation consisting of villous atrophy and crypt hyperplasia. As gluten is the culprit in driving the autoimmune-mediated villous atrophy, its removal from the diet of CD patients leads to symptom relief, restoring of small bowel mucosa, and avoidance of complications. CD has an overall prevalence of about 1% worldwide, with higher rates reported in Northern European countries [1,2].

CD is nowadays widely recognized as a systemic disorder and not only a disease of the small bowel, as many of the adults diagnosed with CD present with extraintestinal manifestations. In fact, the typical presentation with malabsorption syndrome is seen mostly in children and quite rare in adults, who often present with mild, intermittent, and low-intensity digestive symptoms and a wide spectrum of extraintestinal manifestations [3,4,5,6].

The extraintestinal features of CD include a wide range of rheumatologic, neurologic, hematologic, endocrine, metabolic, and dermatologic manifestations [6,7,8,9]. Among them, hematologic findings are one of the most frequent presentations, and sometimes, they can represent the sole manifestation of the disease [10]. In this setting, a high index of suspicion for CD is needed in patients with unexplained, isolated hematological abnormalities, and this depends on better awareness among physicians of general medicine-related specialties [11].

The hematological features of CD include a variety of conditions—anemia, platelet alterations (thrombocytopenia/thrombocytosis), hemorrhagic or thrombotic events, IgA deficiency, hyposplenism, and the fearful lymphoma (Table 1) [12,13].

A high frequency of hematologic alterations (84%) has been reported in CD patients ever since decades ago [14]. Still, there is a high burden of missed CD cases and significant diagnostic delay in frequent clinical situations, such as chronic, unresponsive iron-deficiency anemia. Better recognition of the hematologic findings could be a window of opportunity to increase the diagnostic rate of CD, which is known to be severely underdiagnosed [15]. Although currently available guidelines from the American College of Gastroenterology (ACG), British Society of Gastroenterology (BSG), European Society for Pediatric Gastroenterology, Hepatology, and Nutrition (ESPGHAN), and European Society for the Study of Coeliac Disease (ESsCD) [16,17,18,19] approach some of these hematological features of CD, others are not very well reported.

Our aim was to perform a review of recent literature data regarding hematologic manifestations of CD and their management. For this purpose, we performed a literature search on two databases—PubMed and Embase—from 2010 onwards, using the MESH term "celiac disease" and several keywords referring to the associated hematological features: “hematology”, “anemia”, “thrombocytosis”, “thrombocytopenia”, “hemorrhage”, “thrombosis”, “coagulation”, “IgA deficiency”, “spleen”, and “lymphoma”. Articles identified from this search strategy were checked for access to abstract in English and then further evaluated for relevance to the topic. Clinically significant full-text articles were selected for inclusion in this review; also, references of selected articles were further checked for additional possible meaningful articles, which were not identified by the initial search.

In this review, updated knowledge regarding hematologic manifestations of CD is summarized in accordance with recent data published in the literature.

## 2. Anemia

Anemia in CD patients is multifactorial in etiology; however, iron-deficiency anemia (IDA) is the most common reported [20]. Laboratory workup for IDA can reveal anemia, low mean corpuscular volume, low serum iron, low serum ferritin or anisocytosis (increased red blood cell distribution width) [21]. The main mechanism for IDA in CD is related to malabsorption, as the site of iron absorption—the proximal duodenum—is almost always involved [12]. Severity of iron malabsorption seems to be related to the extent of atrophy along the small bowel, as recent data on ultra-short CD (CD limited to the duodenal bulb) have reported lower proportion of ferritin deficiency in this group compared to extensive CD, both in children and adults [22,23]. Interestingly, anemia in CD is not only related to gluten-driven damage of the bowel mucosa, as it was also reported in patients with positive serology before development of atrophy [24]; this reinforces the need for CD testing in IDA patients and early recommendation of a gluten-free diet in these potential CD patients (the so called “celiac trait”) with extraintestinal manifestations [25].

IDA is one of the most frequent extraintestinal presentations of CD and, according to current guidelines, is an indication for CD screening. According to a recent systematic review and meta-analysis, 3.2% of patients with IDA have biopsy-proven CD [26]. Conversely, up to half of newly diagnosed CD patients, both children and adults, have anemia [10,27,28,29]. In this setting, some authors have even proposed routine duodenal biopsies in IDA patients as a case finding strategy for CD, but this has not proven cost-effective [30,31,32]. As such, the first step in evaluating the suspicion of CD in IDA patients remains serological testing [33], as it is currently recommended in guidelines [34].

One of the characteristics of IDA in CD is refractoriness to oral iron supplements [35]. If symptomatic, correction of anemia can be done by intravenous iron; otherwise, it usually restores in parallel with the histological recovery of atrophic mucosa on gluten-free diet [36]. Lack of anemia correction on follow-up visits should prompt for search of other causes (colonoscopy, capsule endoscopy) and evaluation for refractory CD [37].

Sharing the same site of absorption as iron, folate deficiency can also occur in CD, leading to macrocytic (or normocytic when deficits are combined) anemia; additionally, we should take into account that normocytic anemia does not rule out IDA, as up to 40% of patients with IDA have normal mean corpuscular volume [38]. Studies have reported up to one fifth of patients having low folate levels [27].

Vitamin B12 deficiency was considered theoretically to be less common in CD, as its absorption takes place in the terminal ileum, which is infrequently involved. However, studies have reported significant proportions for B12 deficiency also [20,27].

Anemia of chronic disease, defined by anemia with high ferritin levels and inflammatory syndrome, has been also described in CD [39,40]. Associated aplastic anemia has also been reported in isolated cases [41,42,43].

## 3. Hemorrhagic and Thrombotic Events

Hemorrhagic events can be the presenting feature of CD, including cases of celiac crisis with profound malabsorption and coagulation deficits [44]. A recent review of the literature has found only case reports of hemorrhagic events, comprising otorhinolaryngology, digestive, urology, muscular and alveolar bleeding (the latter defining the Lane Hamilton syndrome) [45]. The mechanism behind hemorrhagic diathesis in CD is mainly represented by vitamin K deficiency, while some studies have also theorized mimicry between factor XIII and tissue transglutaminase [45,46]. Management of hemorrhage consists of intravenous vitamin K and GFD, along with specific measures according to bleeding site.

With respect to thrombotic events, they can also be the prime manifestation of CD. Most cases report on venous thrombosis (deep venous thrombosis, pulmonary embolism, cerebral venous thrombosis, intraabdominal thrombosis), while arterial events have been rarely described [12,47,48,49,50]. In addition to case reports, an increased risk of venous thromboembolism has been shown in large cohort studies [51]. Among the proposed mechanisms, hyperhomocystinemia, protein C/S deficiency, high titers of anti-phospholipid antibodies, and platelet abnormalities have been quoted [52,53].

Although rarer than anemia, hemorrhagic/thrombotic events as a manifestation of CD should be acknowledged accordingly, as they can be of significant clinical impact.

## 4. Lymphoma

CD patients are known to be at increased risk for developing malignancies [54]. Among them, lymphoma is the most fearful complication of CD, as it has a dismal prognosis. In a large population-based case-control study, the odds ratio for developing T-cell lymphoma after a prior diagnosis of CD was 35.8 (95% CI 27.1–47.4) [55]. Patients at risk for lymphoma are those with persistent villous atrophy, meaning those with refractory CD. The absolute risk of lymphoma, while increased, remains low—among 1000 patients with CD followed for 10 years, 7 out of 1000 will develop lymphoma, while the risk is 10/1000 in those with persistent villous atrophy and 4/1000 in healing (similar to that of general population) [56]. Management of lymphoma is multimodal oncologic treatment, but prognosis is often poor.

## 5. Hyposplenism and Susceptibility to Infections

Spleen dysfunction with hyposplenism has also been reported in CD patients. Its underlying mechanism seems to be related to antibody deposits in the spleen [57]. On a peripheral blood smear, one can find some characteristic changes of hyposplenism such as Howell–Jolly bodies, acanthocytes, and target cells [13].

Measuring spleen size is of interest in case of suspected/confirmed CD, as some small-sampled studies have linked splenic hypotrophy with CD and other have shown an association of small spleen volume with refractory CD [58,59,60].

Along with the changes in size, functional hyposplenism is of importance in CD patients, as it can lead to thrombocytosis and susceptibility to infections, especially encapsulated bacteria (*Streptococcus pneumoniae, Haemophilus influenzae, Neisseria meningitidis*) [13]. Immunization against these bacteria should be recommended in CD patients [61,62].

Susceptibility to infections is not only related to hyposplenism, as other factors may also contribute—malnutrition, vitamin D deficiency, altered mucosal permeability and gut microbiota. Increased rates of infections in CD patients have been reported for influenza, herpes zoster, pneumonia, tuberculosis, and Clostridium difficile [63,64,65,66]. However, the risk of infections requiring hospitalization does not seem to be influenced by mucosal healing [67].

## 6. IgA Deficiency

There is a strong association between CD and IgA deficiency, meaning that 2%–3% of CD patients have IgA deficiency and about 8% of individuals with IgA deficiency have CD [13]. Several clinical consequences arise: First, there is the susceptibility to develop other small-bowel diseases such as inflammatory bowel disease or parasite infections (Giardiasis for example, which can histologically mimic CD), then there is the issue regarding diagnosis of CD in these patients, as IgA-based serology can lead to false-negative results (for this reason testing for suspicion of CD includes total serum IgA dosing or both IgA and IgG-based serology), and last, there is a risk of serious transfusion reactions in patients with anti-IgA antibodies [68,69].

## 7. Conclusions

While classical presentations of CD with typical malabsorption syndrome are becoming exceptional, extraintestinal forms are now considered the predominant ones. Among the wide range of extraintestinal features, hematologic-related ones are quite frequent, and they can be the sole manifestation of the disease. IDA is the most frequent hematologic feature of CD, and screening for CD should not be missed in patients with unexplained and refractory to iron-supplementation IDA. Earlier markers of iron-deficiency (alteration in hematological indices of red blood cells) and also changes in platelet numbers should also prompt for testing in a suggestive clinical setting. Hemorrhagic or thrombotic events, otherwise unexplained, can also be the presenting feature of CD. Not least, IgA deficiency and evidence of small-bowel lymphoma should prompt for CD testing. A diagnosis of CD should be always kept in mind in front of a patient with unexplained hematologic abnormalities.

## Figures and Tables

**Table 1:** Hematologic manifestations of celiac disease (CD).

Hematologic Feature	Frequency	Proposed Mechanism
Anemia	Common	Most frequently iron-deficiency, but may be also due to folate, B12 or copper deficiency
Thrombocytopenia	Rare	Autoimmunity
Thrombocytosis	Relatively common	Iron-deficiency, hyposplenism
Hemorrhagic events	Rare	Vitamin K deficiency
Thrombotic events	Rare	Hyperhomocystinemia, elevated levels of other procoagulants, protein C/S deficiency
Hyposplenism	Common	Autoimmunity
IgA deficiency	Relatively common	Associated conditions
Lymphoma	Rare	Refractory CD
